# Data to diagnosis in global health: a 3P approach

**DOI:** 10.1186/s12911-018-0658-y

**Published:** 2018-09-04

**Authors:** Rahul Krishnan Pathinarupothi, P. Durga, Ekanath Srihari Rangan

**Affiliations:** 1 Amrita Center for Wireless Networks & Applications (AmritaWNA), Amrita School of Engineering, Amritapuri, Amrita Vishwa Vidyapeetham, India; 2 School of Medicine, Amrita Institute of Medical Science, Cochin, Amrita Vishwa Vidyapeetham, India

**Keywords:** Precision medicine, Medical informatics, Personalized healthcare, Motif summarization

## Abstract

**Background:**

With connected medical devices fast becoming ubiquitous in healthcare monitoring there is a deluge of data coming from multiple body-attached sensors. Transforming this flood of data into effective and efficient diagnosis is a major challenge.

**Methods:**

To address this challenge, we present a 3P approach: personalized patient monitoring, precision diagnostics, and preventive criticality alerts. In a collaborative work with doctors, we present the design, development, and testing of a healthcare data analytics and communication framework that we call RASPRO (Rapid Active Summarization for effective PROgnosis). The heart of RASPRO is Physician Assist Filters (PAF) that transform unwieldy multi-sensor time series data into summarized patient/disease specific trends in steps of progressive precision as demanded by the doctor for patient’s personalized condition at hand and help in identifying and subsequently predictively alerting the onset of critical conditions. The output of PAFs is a clinically useful, yet extremely succinct summary of a patient’s medical condition, represented as a motif, which could be sent to remote doctors even over SMS, reducing the need for data bandwidths. We evaluate the clinical validity of these techniques using SVM machine learning models measuring both the predictive power and its ability to classify disease condition. We used more than 16,000 min of patient data (*N*=70) from the openly available MIMIC II database for conducting these experiments. Furthermore, we also report the clinical utility of the system through doctor feedback from a large super-speciality hospital in India.

**Results:**

The results show that the RASPRO motifs perform as well as (and in many cases better than) raw time series data. In addition, we also see improvement in diagnostic performance using optimized sensor severity threshold ranges set using the personalization PAF severity quantizer.

**Conclusion:**

The RASPRO-PAF system and the associated techniques are found to be useful in many healthcare applications, especially in remote patient monitoring. The personalization, precision, and prevention PAFs presented in the paper successfully shows remarkable performance in satisfying the goals of 3Ps, thereby providing the advantages of three A’s: availability, affordability, and accessibility in the global health scenario.

**Electronic supplementary material:**

The online version of this article (10.1186/s12911-018-0658-y) contains supplementary material, which is available to authorized users.

## Background

Precision medicine and personalized healthcare are fast gaining wide research interest as well as initial acceptance among the medical community. This is facilitated by the availability of ubiquitous data sources such as wearable sensors, smartphones, and IoT (Internet of Things), along with machine learning and large-scale data analytics tools, resulting in promising outcomes in some of the niche medical domains. Our research particularly focuses on introducing the 3 Ps: precision, personalization, and preventive diagnosis in remote healthcare monitoring of patients, especially in a global health scenario. In our system, patients in remote areas use wearable devices to capture their vital parameters such as blood pressure (BP), blood glucose, oxygen saturation (SpO2), electro cardiographs (ECG) etc., and transmit them to doctors in tertiary care hospitals, who in turn are expected to suggest suitably needed timely interventions. While deploying our system in the highly populous region of southern India, we found that, although this promises to provide hitherto unavailable healthcare services to critically ill and aging population particularly in the developing world, there are significant roadblocks in our expectation that doctors embrace this new paradigm in handling patients. The doctors, who are already overloaded, feel even more overwhelmed by the voluminous data being flooded from remote patients’ sensors. Furthermore, interpreting such multi-parameter data pouring in simultaneously from a multitude of remote patients is time-consuming and soon transforms into an unmanageable deluge.

### Approach

In this paper, we propose novel approaches to transform data into diagnosis. As a collaborative work between our researchers and clinicians in one of the largest super-specialty hospitals in India (Amrita Institute of Medical Sciences - AIMS), we developed physician assist filters (PAFs) that are designed to transform unwieldy time series sensor data into summarized patient/disease specific trends in steps of progressive precision as demanded by the doctor for patient’s personalized condition at hand, and help in identifying and subsequently predictively alerting the onset of critical conditions. Together with the communication network and data transmission architecture, this new framework that we have designed, developed, and successfully deployed is called RASPRO (Rapid Active Summarization for effective PROgnosis). The RASPRO framework was first introduced in [1].

### Related work

We begin by analyzing the existing systems that simply generate alerts every time one or more sensors cross the abnormality thresholds. Due to the humongous volume of such alerts, they are difficult to manage even in the case of hospital in-patient settings, let alone for a much larger number of remotely monitored patients. Starting from some of the initial attempts reported in [2], to more recent works such as [3–5], and [6], the severity detection and alert generation is typically based either on predefined thresholds, or based on training of thresholds using machine learning followed by online classification of multi-sensor data. Very similar techniques of machine learning have also been used in fall detection [7, 8]. Hristoskova et al. [9] propose another system wherein patient conditions are mapped to medical conditions using ontology-driven methods, and alerts generated based on corresponding risk stratification. Even though there has been noticeable success in detection and diagnosis of specific disease conditions, most of these works have not explored the opportunity for personalized and precision diagnosis. In an extensive review of Big Data for Health, Andreu-Perez et al. [10] specifically emphasize on the opportunity for stratified patient management and personalized health diagnostics citing examples of customized blood pressure management [11]. More specifically, Bates et al. [12] discuss the utility of using analytics to predict adverse events, which could reduce the associated morbidity and mortality rates. Furthermore, Bates et al. [12] argue that patient data analytics based on early information supplied to the hospital prior to admission can result in better management of staffing and other hospital resources. One of the recent works in personalized criticality detection is reported in [13], which propose an analytical unit in which the Improved Particle Swarm Optimization (IPSO) algorithm is used to arrive at patient-specific threat ranges. To improve precision in diagnosis we also need to arrive at a balance between a completely automated system on one hand, and physician assist systems on the other. Celler et al. [14] propose a balanced approach wherein sophisticated analytics are presented to physicians who in turn identify the changes and decide on the diagnosis. This is also supported by many results including that reported in [6], wherein domain knowledge based method performed as well as other trained machine learning models. These arguments and results provide further impetus for personalized, precision, and preventive diagnostic techniques that are amenable to physician interventions.

## Methods

**Fig. 1:**
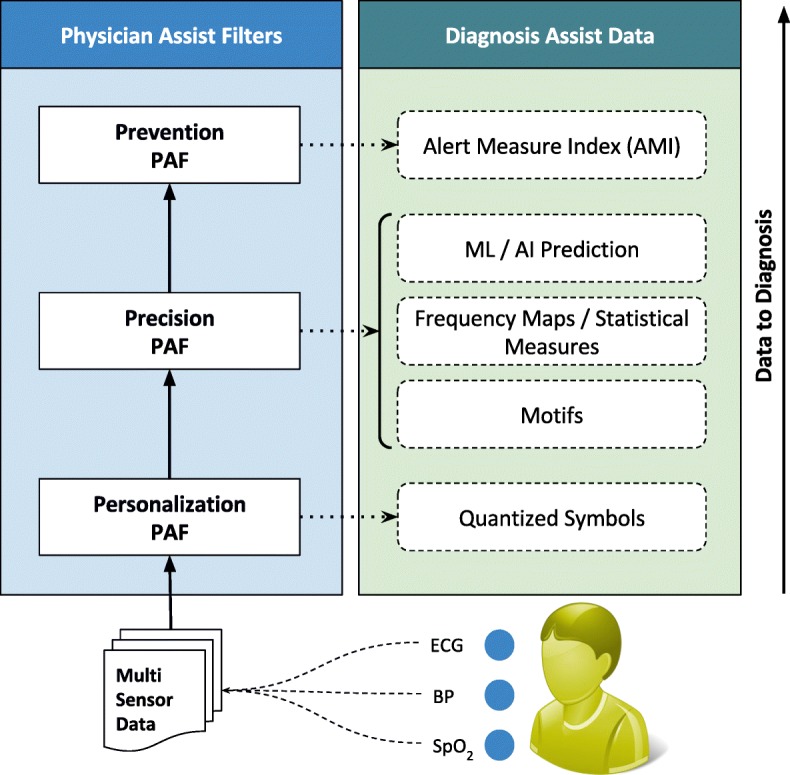
RASPRO-PAF Framework The architecture shows the RASPRO-PAF framework which progressively converts the raw multi-sensor data into quantized symbols, helpful motifs, diagnostic predictions, and critical alerts

The first significant improvement that we bring into bear is the quantization of every remotely sensed parameter based on its own customized severity boundaries. Sequential time windows of such quantized values are examined for dominant appearances of normalcies or abnormalities, as the case may be, and motifs corresponding to them are extracted. Using factors set by doctors, the system then transforms these motifs by generating interventional time alerts as per clinically prescribed protocols. Both the alerts and motifs are amenable to rapid transmission to doctors, even as SMSs on bare minimum bandwidth starved wide area wireless networks. This results in the generation of more clinically relevant critical information, along with a drastic reduction in reporting every minor aberrational data that may not be indicative of any serious condition, after all. The system does not stop here. The attending doctors, when they view the alerts and/or motifs, have the luxury to request Detailed Data on Demand (dubbed DD-on-D), upon which the next level of detail in the data is transmitted. This level of detail could be a straightforward frequency map of normal and abnormal values, or much more intelligent machine learning classifications in the case of proven disease conditions. The heart of our system is a framework called RASPRO (see Fig. 1) consisting of Physician Assist Filters (PAFs) that, in going from data to diagnosis, implement the 3 Ps: Precision, Personalization, and Prevention. In the following sections we describe each of these 3Ps in detail.

## Personalization PAF

Due to the distributed data gathering and processing architecture, there is an opportunity to enhance personalization in diagnosis and treatment. The first component in the RASPRO framework, the personalization PAF takes the form of a patient and disease condition specific severity quantizer that converts raw sensor values to a series of clinically relevant severity symbols.

### Adaptive quantization

**Fig. 2:**
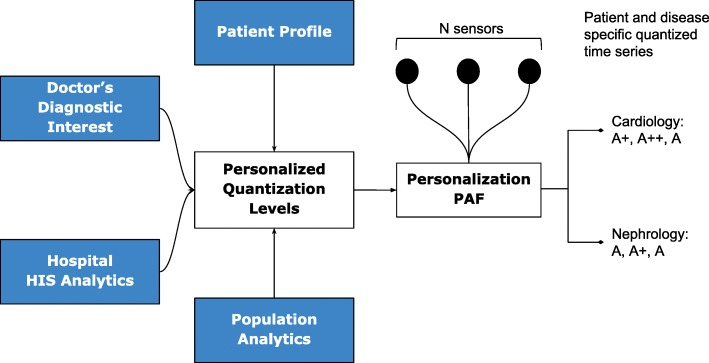
Personalized Quantization Quantization of sensor data is based on multiple severity categorization criteria, resulting in the generation of patient and disease specific quantized values

In general, let us consider N body sensors, *S*_1_,*S*_2_,…,*S*_*N*_ with varying sensing frequencies *f*_1_,*f*_2_,…,*f*_*N*_. The raw time series values from these sensors are converted to discrete severity level symbols by the quantizer. The number of severity levels *L*_*i*_ for a sensor *S*_*i*_ can be set based on the sensor and many other factors. We assume that different vital parameter sensors have different number of severity levels, and hence *L*_1_, say the number of severity levels for a blood pressure sensor, could be equal to five, whereas, *L*_2_ (say oxygen saturation levels) could be equal to seven. In our symbolic notation, the clinically accepted normal values are assigned the symbol **A**, while above-normal values are assigned with progressive degrees of severity as *A+, A++* etc., while that of sub-normal values are assigned *A-, A−−* etc.; the number of **“+”** and **“-”** symbols representing degree of normal and subnormal severities respectively. Figure 2 depicts how various severity levels are arrived at in Personalization PAF severity quantizer.

The quantized severity symbols are arranged into a Patient Specific Matrix (PSM) of N rows and W columns, where N is the total number of sensors being observed, and W is a time window in which the data is summarized. The value of W can be set by a physician or automatically derived based on the risk perception of that particular patient.

### Personalization

The quantization breadth are decided by doctors based on the patient profile (or history), doctor’s diagnostic interest (for instance, a cardiologist may assign severity ranges differently from that of a nephrologist), severity ranges as suggested by using analytics on local hospital information system (HIS), and also based on population analytics across multiple HIS spanning multiple hospitals or even from publicly available databases such as PhysioNet [15]. Together, this approach gives ample flexibility in achieving customization in inter-patient, inter-disease, intra-patient, inter-specialty diagnosis from multi-sensor data.

## Precision PAF

Whereas in most other applications, precision directly translates into great detail in data, in remote health monitoring, precision cannot come at a cost of voluminous data presentation to the doctor. Compactness has to be retained. We have developed a step-wise refinement process for precision, which is delivered on demand to the attending doctor. Step 1 is “Consensus Motifs (CM)”, Step 2 is a collection of statistical parameters including severity frequency maps (SFMs), and Step 3 is Machine Learning (ML). In the first step, motifs corresponding to commonly seen normalcies and abnormalities in the severity symbols series are extracted. The outcome of this is two severity summaries: (1) the most frequent trend in sensor data that we call as consensus normal motif (CNM), and (2) the most frequently occurring abnormality that we term as consensus abnormality motif (CAM), to construct which, we use the following building blocks.

**Candidate Symbol**, *α*[*p*] is the p-th quantized severity symbol in a row of the PSM, *α*[1],*α*[2],…,*α*[*p*],…,*α*[*W*].

**Normal Symbol**, *α*_*NORM*_ is a candidate symbol that represents the normal level and its value is equal to **“A”** for every sensor.

Now, let the set ***C***_***n***_ denote all the candidate symbols in a **W**-long observation window, corresponding to *n*-th sensor in the PSM. However, we have dropped the subscript *n* for better clarity of discussion.

$$C = \{\alpha[1], \alpha[2], \ldots, \alpha[p], \ldots, \alpha[W]\}. $$

Let *σ*[*p*] denote the sum of hamming distances of *α*[*p*] from all other candidate symbols in C such that:

$$\sigma[p] = \Sigma_{i=1}^{W}D(\alpha[p],\alpha[i]) $$

where, *D*(*α*[*p*],*α*[*i*]) is the hamming distance of *α*[*p*] from *α*[*i*]. Here, we assume that the hamming distance between neighboring severity levels (say, A and A+) is 1. We define a set **H** of all *σ*’s such that:

$$H = \{\sigma[1], \sigma[2], \ldots, \sigma[p], \ldots, \sigma[W]\}. $$

**Consensus Normal Symbol**, *α*_*CNS*_[*C*] is defined as a candidate symbol among all the symbols in **C** that satisfies the following two conditions: (1) its hamming distance from the normal symbol, denoted as *D*(*α*_*CNS*_[*C*],*α*_*NORM*_), is less than a sensor specific near-normal severity threshold *S*[*n*]_*THRESH*_, and (2) its sum of hamming distances from all other candidate symbols in **C** is the minimum. This is formulated as:

1 $$ {{}\begin{aligned} \alpha_{CNS}[\!C] &= \{\alpha[p] : D\left(\alpha[p],\alpha_{NORM}\right)\! < S[n]_{THRESH}\\ &\qquad and ~\sigma[\!p] ~is ~the ~lowest ~such ~candidate ~in ~H\}. \end{aligned}}  $$

**Consensus Abnormality Symbol**, *α*_*CAS*_[*C*] is defined as a candidate symbol in **C** that satisfies the following two conditions: its hamming distance from normal symbol, *D*(*α*_*CAS*_[*C*],*α*_*NORM*_) is greater than or equal to a sensor specific near-normal severity threshold *S*[*n*]_*THRESH*_ and the sum of hamming distances from all other candidate symbols in **C** is the minimum. This is formulated as:

2 $$ {{}\begin{aligned} \alpha_{CAS}[\!C] &= \{\alpha[p] : D(\alpha[p],\alpha_{NORM}) \geq S[n]_{THRESH}\\ &\qquad and ~\sigma[p] ~is ~the ~lowest ~such ~candidate ~in ~H\}. \end{aligned}}  $$

Defining upper and lower bounds for the deviations of *α*[*p*] from *α*_*NORM*_ using the *S*[*n*]_*THRESH*_ variable ensures that the doctors can choose to differentiate “near-normal” severity levels from those which are critical in nature, providing for increased personalization and precision in diagnosis.

**Consensus Normal Motif**, *μ*_*CNM*_[*P*] is an ordered sequence of consensus normal symbols belonging to **N** rows in the PSM of a patient **P**, and is represented as <*α*_*CNS*_[*C*_1_],*α*_*CNS*_[*C*_2_],…,*α*_*CNS*_[*C*_*N*_]>. The *n*-th consensus normal symbol *α*_*CNS*_[*C*_*n*_] in *μ*_*CNM*_[*P*] can be indexed as *μ*_*CNM*_[*P*][*n*].

**Consensus Abnormality Motif**, *μ*_*CAM*_[*P*] is an ordered sequence of consensus abnormality symbols belonging to **N** rows in the PSM of patient **P**, which is represented as <*α*_*CAS*_[*C*_1_],*α*_*CAS*_[*C*_2_],…,*α*_*CAS*_[*C*_*N*_]>. The *n*-th consensus abnormality symbol *α*_*CAS*_[*C*_*n*_] in *μ*_*CAM*_[*P*] can be indexed as *μ*_*CAM*_[*P*][*n*].

**Fig. 3:**
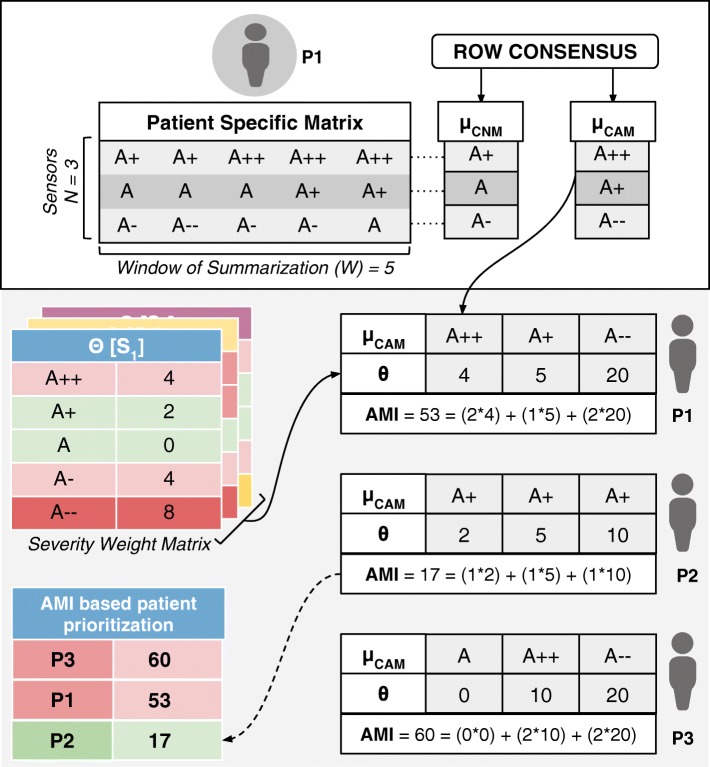
RASPRO Severity detection, summarization, and AMI calculated using CAMs and sensor specific severity weight matrix. It also shows an AMI based patient prioritization table that can help physicians in attending to the neediest patient

To reiterate in the above formulation, each row of a PSM is considered as an observation window set C (corresponding to a summarization time window W) to find the corresponding consensus symbols, *α*_*CNS*_[*C*] and *α*_*CAS*_[*C*]. The sequence of these symbols over the N rows in a PSM, form column vector motifs *μ*_*CNM*_[*P*] and *μ*_*CAM*_[*P*] (refer to Fig. 3).

In subsequent steps of Precision PAF, the system generates a frequency map that shows how frequently different multi-sensor parameters have crossed the personalized severity thresholds. Finally, the motif time series is further used as input to proven deep learning (DL) and machine learning (ML) techniques such as Long Short Term Memory (LSTM) recurrent neural networks (RNN) [16] or Support Vector Machines (SVM) [17] that could help the doctors in diagnosis. In the next section, we use the above consensus motifs for alert generation to aid in criticality prevention.

## Prevention PAF

Implemented as an alert generation technique that uses simple or complex mathematical models, to calculate the amount of time available to the physicians for effective intervention, the Prevention PAF is amenable to changes based on patient, disease, and physician’s diagnostic interest. The output of the Prevention PAF is an alert measure index (AMI) that is used to prioritize the patients based on their urgency for physicians’ interventional attention.

Each severity symbol in a motif also communicates how much time is available with the doctor for deciding an intervention (any if needed). Hence, for each sensor *S*_1_, *S*_2_, …, *S*_*N*_ and its corresponding severity symbol *α* in *μ*_*CNM*_[*P*] and *μ*_*CAM*_[*P*] (where *α* could be A, A+, A-, etc.) we associate it with a corresponding medically accepted intervention time *δ*[*S*_*n*_][*α*]. Across different sensors *S*_*n*_ for a patient *P*, let us consider *θ*[*S*_*n*_][*α*] as a sensor and severity symbol indexed matrix of weights derived from interventional time using the following relationship:

3 $$ \theta\left[S_{n}\right][\!\alpha] = \frac{K_{P}}{\delta\left[S_{n}\right][\!\alpha]}  $$

In Eq. 3, the constant *K*_*P*_ can be set by the physician considering the context of patient’s health condition (including historical medical records and specific sensitivities and vulnerabilities documented therein) or derived through machine learning techniques. Equation 3 may be substituted by more complex equations for progressively complicated disease conditions.

At the end of each observation time-window W, for every patient *P*, we also define an aggregate criticality alert score, called the Alert Measure Index (AMI), which is calculated as:

4 $$ {{}\begin{aligned} AMI = \Sigma_{n=1}^{N} \left(\theta\left[S_{n}\right]\left[\mu_{CAM}[P][\!n]\right]\right) * num\left(\mu_{CAM}[\!P][\!n]\!\right) \end{aligned}}  $$

wherein, each severity quantized symbol in the *μ*_*CAM*_[*P*] of the n-th sensor is converted into a numerical value (e.g., *A*± is assigned 1, *A*++ or *A*−− is assigned 2) using *num*(*μ*_*CAM*_[*P*][*n*]), and scales it up by the sensor-severity specific weight *θ*[*S*_*n*_][*α*] (defined in Eq. 3). The resulting AMI is indicative of the immediacy of patient priority for physician’s consultative attention. The process of motif detection, AMI calculation, and patient prioritization is summarized in Fig. 3. The data used to arrive at the AMI scores could be other statistical parameters (such as frequency maps) or machine learning prediction scores. Also, the technique for calculating the score may also be based on predefined simple mathematical models, or complex machine learning algorithms.

## Clinical relevance and validation

In October 2016, the RASPRO framework was introduced to doctors in multiple specialties in our super-specialty hospital, wherein they validated its clinical deployment applications. We present some of the specific clinical scenarios that emerged from this pilot study.

### Cardiology

The electrocardiogram is a potential indicator of cardiac events and can be exploited for personalized and precision diagnosis by varying the parametric thresholds and summarization window, based on patient profile/disease condition and associated factors. For instance taking into account the disease condition, a 3mm depression in the ST segment would be graded as A++ for an active patient having exertion related chest pain, indicating cardiac ischemia, whereas the same if occurred in a patient at rest, would be graded as A+++ with limited time of intervention (30 min), indicating cardiac muscle death. To extend the spectrum of diseases that ST segment depression would cover, a chronic hypertensive with left ventricular hypertrophy of the heart (and no chest pain) would also presumably have a continuous 3mm dip in the ST segment which does not require any interventional attention, and hence, would be graded as A/A+ (near normal) by the severity quantizer. Next, taking into account the patient profile, in sedentary workers, aged above 45 having smoking habit, with high cholesterol levels, and other associated risks the thresholds will be low (A+, A++, and A+++ would be assigned to 1–2mm, 2–3mm, and above 3mm ST depression respectively) while in highly active but risk patients with age less than 45, and no previous associated history, the levels will be high (A+, A++, and A+++ would correspondingly be assigned to 2–3mm, 3–3.5mm, and above 3.5mm respectively). Also, in the former case the summarization window W (capturing how long ST depression sustains) would be 3–4 min (more critical), whereas in the latter it would be 7–9 min.

### Pulmonology

Simple but vital parameters such as oxygen saturation levels in the body (SpO2), BP, heart rate variability (HRV), and respiratory rate variability (RRV), present in unique combinations, would facilitate differentiating between benign diseases such as interstitial lung disease/sleep apnea for which the thresholds for alert (set through the interventional time constant *K*_*P*_) will be fairly high, and emergencies such as pulmonary edema/pulmonary embolism (blood clot in an artery in the lung) for which the thresholds will be kept low if any of the predisposing factors such as left heart failure, pulmonary hypertension, prolonged immobilization, pregnancy, etc. are present. Hence, the physician would preset these combinations of vitals to be looked for as sequence of symbols in the CAM. Since the number of parameters that could be picked up to indicate disease are a few, it is pertinent that stepwise precision techniques such as machine learning algorithms be used for distinguishing between closely mimicking conditions. To quote as an example is obstructive sleep apnea and chronic obstructive pulmonary disease, both of which would show similar trends in SpO2, HRV, RRV and BP. In a trial that was conducted at our hospital, we were able to achieve 99% precision in diagnosing sleep apnea from HRV using deep learning algorithm called Long short-term memory recurrent neural networks (LSTM-RNN) and is reported in one of our previous works [16]. The algorithm evaluation was done using the multi-sensor patient data from the Physionet Challenge 2000 [18], which contained annotated data from 35 patients who underwent overnight sleep study.

### Neurology

One of the early markers of autonomic neuropathy in epileptic patients is the discrepancy between the BP and the pulse rate of the patients. In this scenario, the severity levels of BP and pulse rate would be set accordingly (as a combination) to alert the practitioner. Suppose S1 is BP and S2 is heart rate sensor respectively. Let us say for the patient P1, *μ*_*CAM*_[*P*1]=<*A*−−,*A*>, and for patient P2, *μ*_*CAM*_[*P*]=<*A*−,*A*+>. In both cases, the diagnosis, alert level, and treatment vary because P1 has BP decline with no change in heart rate (critical), while P2 has a compensatory increase in heart rate, which indicates good autonomic function.

Though these are representative clinical scenarios, we found wide agreement among the doctors from other specialties too that personalization, step-wise precision, and prevention introduced through the RASPRO framework is of high utility in remote monitoring and critical alert generation.

## Results and discussion

In order to quantitatively evaluate the effectiveness of RASPRO, we measure both the diagnostic ability as well as the preventive predictive power of this technique. We formulate three hypotheses and evaluate the effectiveness of RASPRO in satisfying these:

- **Precision Hypothesis:** RASPRO consensus motif time series can replace raw sensor data time series for the task of identification/classification of specific disease conditions.
- **Prevention Hypothesis:** RAPSRO based consensus motifs can predict future disease condition with as much accuracy as raw sensor data time series.
- **Personalization Hypothesis:** There exists an inter-patient variability in severity levels and summarization frequencies, which if optimized individually can result in better accuracy in predicting/classifying a specific disease condition.

By assessing the validity of the first hypotheses we aim to evaluate the extent to which RASPRO motifs can provide precision in diagnostics. The second hypothesis evaluates the utility of RASPRO as a tool for predictive analytics in critical conditions; while the third hypothesis help us understand if there exists a case for personalization in disease discovery and prediction.

### Dataset

The first step to evaluate these hypotheses is to identify datasets that are extensive, long term and critically significant. We used large time series dataset from MIMIC II [19] database, which contains multiple body sensor values from over 20,000 ICU patients. This dataset consists of ECG, ABP (Arterial Blood Pressure), Heart Rate (HR), Non-obtrusive BP (NBP), SpO2, Mean Arterial BP (MAP), and other vital signs. From this, we selected a curated set of patient and control group data that contained a long time series data followed by a critical event. We selected patients with Acute Hypotensive Episodes (AHE), which is a potentially fatal condition, found quite common in ICUs as well as caused due to postural hypotension. An AHE event is analytically identified as when MAP measurements remain below 60 mmHg for more than 30 min. This is a potentially fatal event and requires immediate intervention. We also made sure that the dataset provides uninterrupted MAP signal with a minimum sampling rate of 1 per minute, over at least 3 h for both the event-patients as well as the control group. We selected a group of 35 patients (called group H) who had AHE during some time during their stay in ICU, and another 35 patients (called group G) who did not have AHE during their ICU stay. This dataset was selected from the PhysioNet [15] challenge 2009 [20]. The H dataset also had a time marker *t*_0_, after which AHE occurred in that patient within a one-hour window. Since the data was obtained from publicly available sources, we did not require getting prior approval of IRB for this work.

### Evaluating precision hypothesis

The first task is to measure the replaceability of the original time series data with the quantized symbols and consensus motifs. To evaluate this, the H and G group time series data comprising of Mean Arterial Pressure (MAP), of length 60 min after *t*_0_ are modeled as feature vectors of length 60. These vectors are called original time series (OTS), and are used for training an SVM model for classifying the data as having AHE or not. The vectors belonging to AHE were labelled as H, and G otherwise. After using OTS, we then generate quantized time series (QTS) vectors with different quantization breadth. The quantization breadth (denoted by B) are varied as 5, 10, 15, and 20. For instance, when B=10, each of the OTS MAP values between 60 mmHg and 50 mmHg are quantized into the same severity symbol, say “A-”, whereas for B=5, the symbol “A-” quantizes all OTS MAP values between 60 mmHg and 55 mmHg. These vectors are used in similar manner to first train and then test the SVM model. Finally, we generate the corresponding motif time series (MTS) for each of the QTS, with varying the summarization time window W as 5, 10, and 15. The value of W corresponds to the time window in which all the severity symbols in the QTS are converted to a single consensus symbol. A comparison of OTS, QTS, and MTS is done using the statistical measure of binary classification, the F-score. An F-score (also called *F*_1_-score) is calculated as:

5 $$ F_{1}score = 2 * \frac{(Precision * Recall)}{(Precision+Recall)}  $$

#### Significant results

**Fig. 4:**
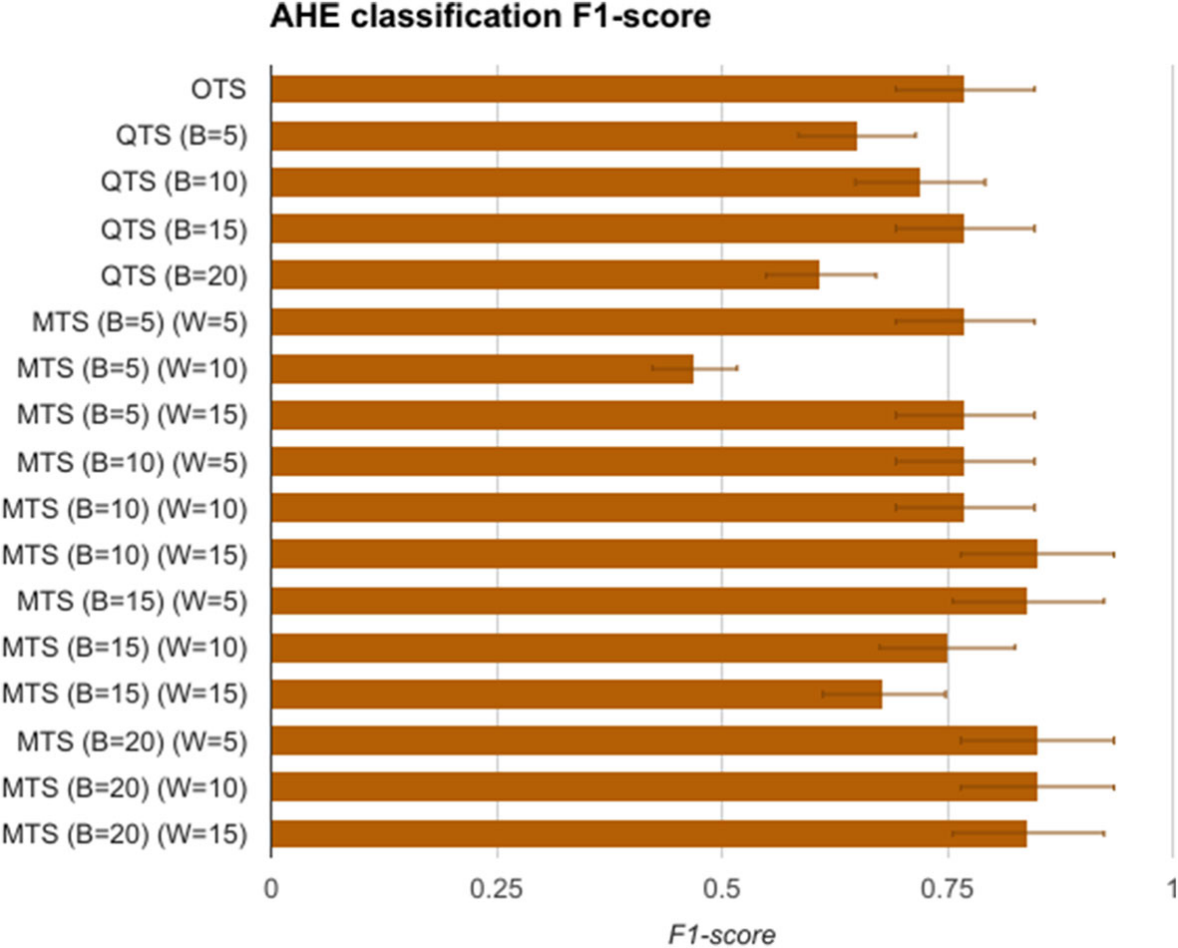
AHE classification F1-score The F1-scores of SVM models trained and tested for classifying the given 60 min of data as AHE or not using OTS, QTS (B=5,10,15,20), and MTS (W=5,10,15). It shows that QTS and MTS with different B and W values are able to classify the AHE signal with F1-scores that are better than one obtained using OTS SVM model

The F1-scores for the SVM models are summarized in Fig. 4. It shows that OTS based SVM model gave an F1-score of 0.76, which is the gold standard that we compare other models with. The QTS and MTS based SVM models were able to perform as well as OTS in most of the cases. Furthermore, MTS with (B=10 and W=15) and (B=20 and W=5,10,15) performed better than the OTS in the classification problem. In fact, these MTS models showed more than 12% better F1-score compared to OTS. These results support the precision hypothesis that motif time series can replace original time series data for the task of identification/classification of specific disease conditions, in this case AHE.

### Evaluating prevention hypothesis

The next evaluation parameter of RASPRO is to identify if a priori motif series could predict future disease condition, and thereby aid in preventive intervention. For this, the H and G group time series data comprising of Mean Arterial Pressure (MAP), of length T minutes prior to *t*_0_ is modeled as a T-long feature vector (OTS). These vectors are used for training (using 70% data, with 5 fold cross validation) and testing (using 30% data) an SVM model for predicting them as AHE or not, where patients belonging to H group are annotated as having AHE and G group patients are annotated otherwise. In effect, we try to classify sensor data prior to AHE event as a predictor for ensuing AHE condition. Since G group data did not have a time marker *t*_0_ we selected a random but continuous time series of length T from each of the G group patients. SVM was selected due to its widely accepted performance in classification problems involving multiple features, although we might obtain comparable results using other classification techniques too.

The backward offset time T (from *t*_0_) is varied as 30, 60, 90, 120, 150, and 180 min as an expanding window. In the next step, the raw feature vectors are quantized using severity quantizer to form a quantized time series (QTS). Once again, the quantization breadth B is varied as 5, 10, 15, and 20. In the third step, the QTS are summarized and motifs extracted to form RASPRO Motif Time Series (MTS), with varying observation time window sizes W: 5, 10 and 15 min. The QTS and MTS are then given as input to train and test the SVM model (one for QTS and another for MTS) for predicting AHE before it’s onset.

#### Significant results

**Fig. 5:**
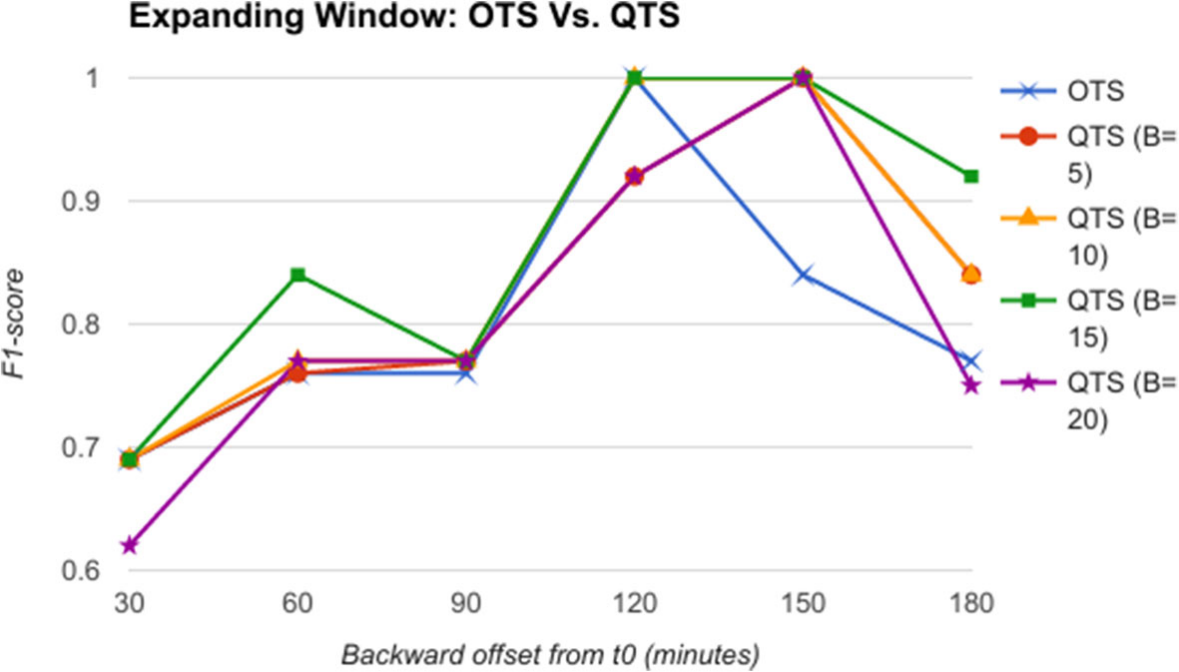
Expanding Window: OTS Vs. QTS Comparison of F1-score of OTS and QTS for classification of AHE using expanding time windows shows better performance of QTS with B=15

**Fig. 6:**
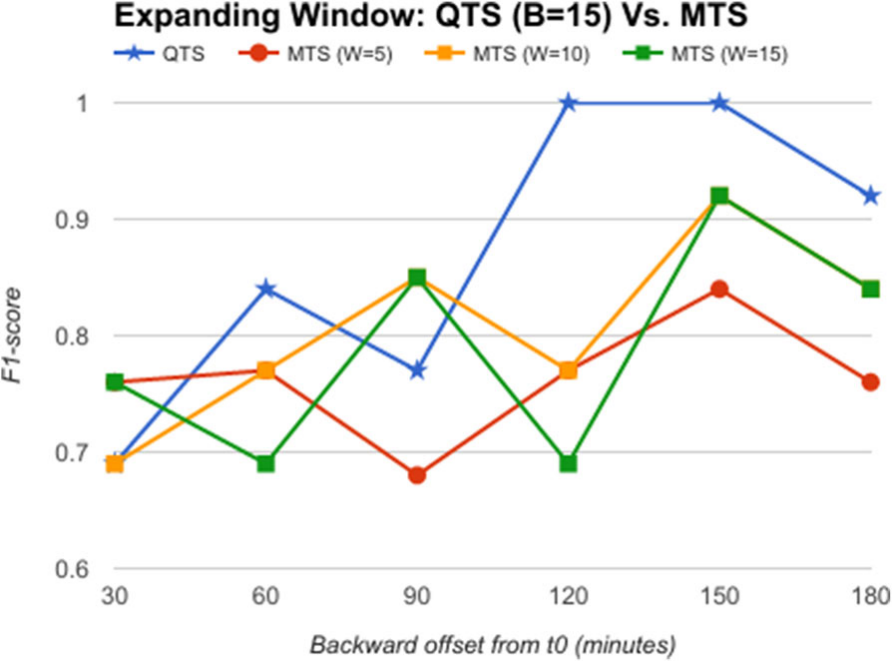
Expanding Window: QTS Vs. MTS Comparison of F1-score of QTS (B=15) and MTS (varying W) for classification of AHE using expanding time windows

**Fig. 7:**
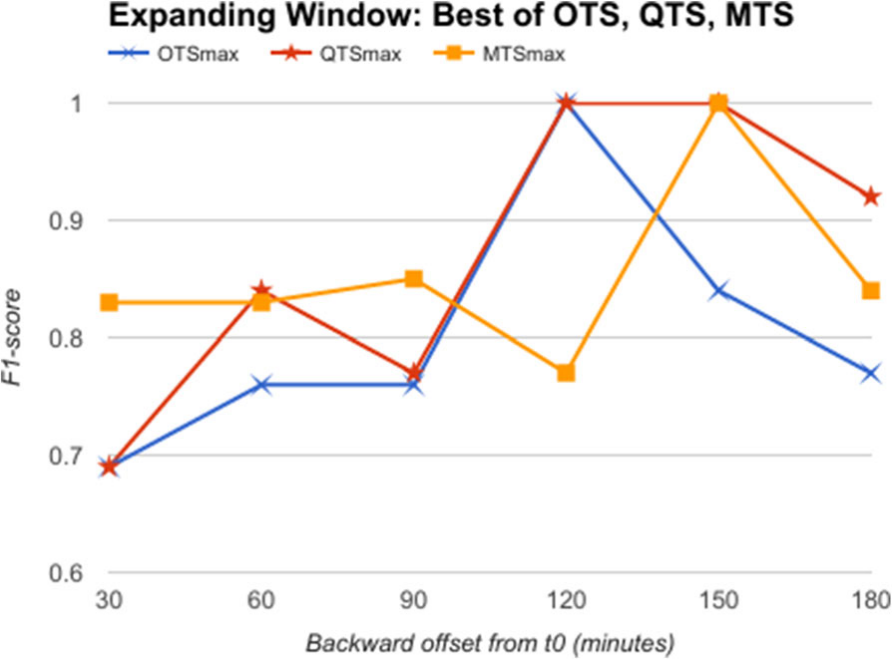
Expanding Window: QTSmax Vs. MTSmax Comparison of F1-score of OTS with QTSmax and MTSmax corresponding to best performing B and W values respectively for classifying AHE using expanding time windows

From the comparative analysis of OTS and QTS (Fig. 5), we observe that QTS with B=15 has better F1-score in comparison to OTS in all the time-offsets T, although the root mean square error (RMSE) between these two series is an insignificant 0.001, pointing to the fact that OTS could be replaced with QTS. We select this QTS (B=15) and then compare it with MTS of varying time windows in Fig. 6. We observe from Fig. 6 that QTS has higher F1-score compared to the best MTS with W=10. However, RMSE between QTS and MTS (W=10 and W=15) is a statistically insignificant value of 0.01, which implies that MTS using W=10 and 15 performs as well as QTS on an average across different time windows. Now, we further compare the OTS against the best performing B and W values corresponding to QTS and MTS respectively, and the results are plotted in Fig. 7. These data points are marked as QTSmax and MTSmax respectively. In Fig. 7, QTSmax and MTSmax show closely similar F1-score with the RMSE as 0.018, which could be considered statistically insignificant.

**Fig. 8:**
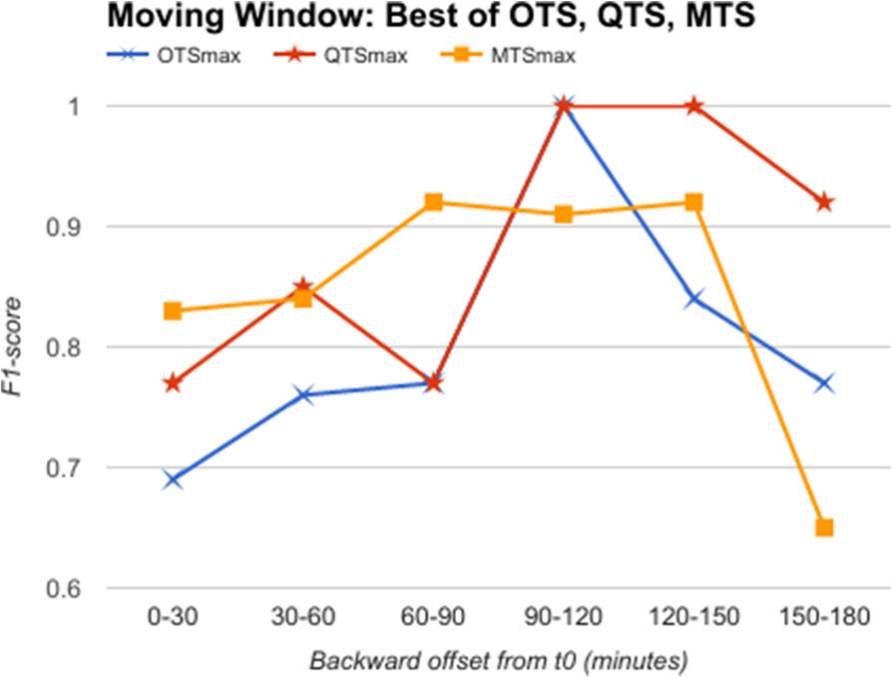
Moving Window: QTSmax Vs. MTSmax Comparison of F1-score of OTS with QTSmax and MTSmax corresponding to best performing B and W values respectively for classifying AHE using moving window of 30 min duration

Going further, we used data from a moving time window of 30 min each, instead of an expanding window. This simulates the situation when we obtain data for 30 min alone and are required to classify it as a predictor for AHE. Here, we do not have the luxury of having data till *t*_0_, as the 30 min slice of data could be from anywhere upto 3 h before *t*_0_. We show in Fig. 8 the comparative analysis of OTS against the best B and W values corresponding to QTS and MTS in the moving window experiment. The results plotted in Fig. 8 show that MTS and QTS perform better than OTS in most of the time intervals, while the RMSE between MTS and QTS is 0.018 on an average. The results comparing QTS with different B values against MTS with different W values are given in Additional files 1, 2, 3, 4 and 5.

From these results, we can conclude that quantized symbols, as well as summarized motifs, are as good as (or better in many cases) compared to raw time series in identifying predictors for AHE, both in expanding and moving windows, thereby supporting our prevention hypothesis.

### Evaluating personalization hypothesis

The third hypothesis aims to find out if there are patient specific custom severity levels, and summarization frequencies, which if optimized could lead to better accuracies in diagnosis. For this, we further analyze our earlier results. We observe from Figs. 7 and 8 that by selecting different severity quantization breadth (B) and through varying the summarization window size (W), we are able to predict the onset of AHE with higher F1-score. This supports an argument for using disease and time-specific B and W values for achieving better accuracy in classification problems. We observe very similar results in Fig. 4, which shows that by choosing optimized W and B values, the machine learning models can perform better in classification problems too. These results further support our third hypothesis, that there exists an opportunity for personalization at least at disease specific and time specific level. Though the above experiments using AHE are only representative of how step-wise precision, personalization, and prevention can be achieved using RASPRO, the practitioners as a whole agree that in wide-ranging scenarios patient-sensor-disease-time specific severity levels need to be defined that is both practical to manage alerts as well as effective in identifying emergencies.

### Global health deployment

These medical benefits of RASPRO framework would contribute directly to fulfill the primary goals of remote health monitoring in global health scenario. We call these as the *3A* benefits in short, which stands for: *availability*, *accessibility*, and *affordability*.

**Fig. 9:**
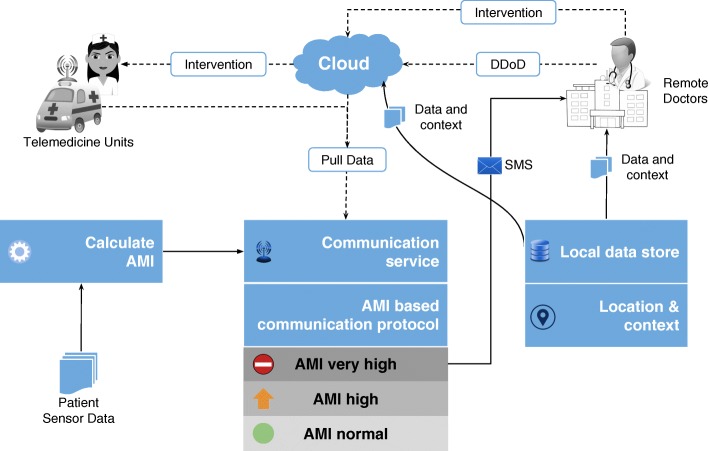
Detailed Data-on-Demand The DD-on-D technique as implemented in RASPRO-PAF framework enables patient’s multi-sensor data to be sent over even SMS to remote doctors, who then initiate emergency intervention through telemedicine units stationed near to the patients location
- *Affordability*: Remote health monitoring combined with timely criticality detection can substantially reduce the healthcare costs, by reducing the number of unnecessary hospital visits and smartly managing the available time of doctors who could focus on the neediest of patients. For instance, in a developing country the patients could be spending anywhere between $4-5 for travelling to the nearest hospital. Combined with the loss of their daily wages due to taking a break from their work, the cost to the patient for a hospital visit could be around $10-20 per day, not including the consultation charges (which ranges between $5-10 per visit). Through an initial survey of the patients visiting the cardiology department in our hospital it was observed that a majority of the patients do not, at the end of examination, have a cardiac disease. These could well have been diagnosed as such using remote monitoring of their vital parameters and hence avoid unnecessary hospital visits. Also, for a majority of revisiting patients, the visits could have been avoided using remote monitoring.

- *Availability*: By enabling the doctors to prioritize their time based on the AMI, we effectively increase the availability of doctors for the neediest of remote patients.
- *Accessibility*: A patient’s summarized health status represented by the consensus motifs could be sent over even bare minimum communication networks (for instance, in the form of SMS). The clinically validated RASPRO motifs would then enable the doctors to use it instead of voluminous raw sensor data for arriving at timely diagnosis. In addition, by providing step-wise precision through detailed data-on-demand (DD-on-D), the doctors can choose to get more data if needed. Together, these techniques, as illustrated in Fig. 9, increase the accessibility of patients’ to quality and critical remote healthcare services.

**Fig. 10:**
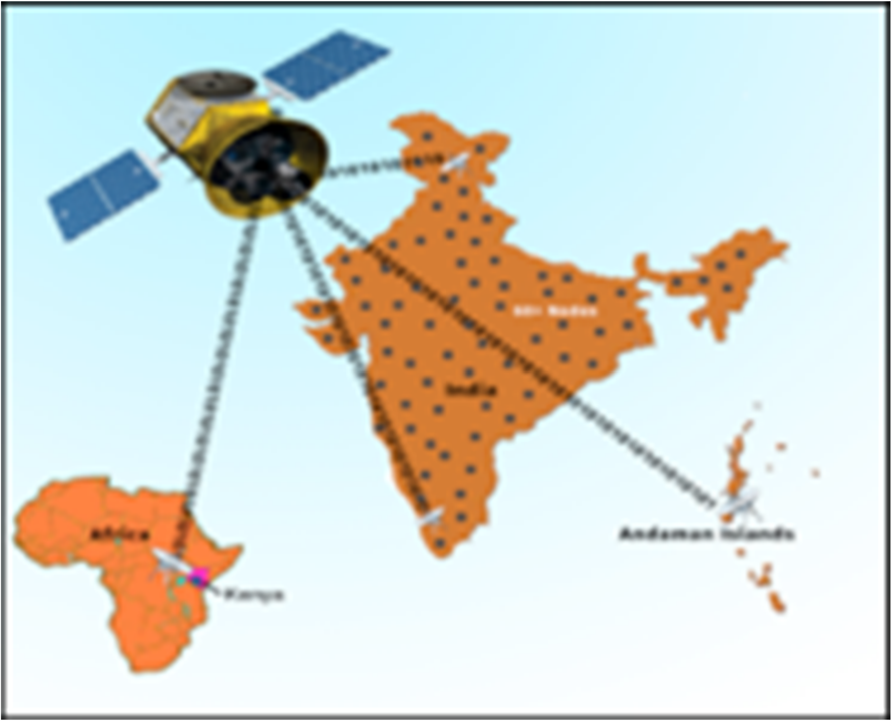
Global Health Deployment The RASPRO-PAF system is being readied for deployment using the telemedicine network of AIMS hospital, which has more than 45 remote nodes spread across India and Africa, connected through satellite network

These advantages would help bring quality healthcare to millions of people who are currently under-served in the global health scenario. We are readying for large-scale deployment of the RASPRO framework including the 3P RASPRO-PAF analytical tools using a network of more than 45+ telemedicine nodes (as shown in Fig. 10) and remote health centers across the Indian sub-continent, which are connected to the AIMS hospital.

**Practitioner education** is one of the key challenges in global deployment of any new data analytics technique. To ensure usability of the system, we have involved the doctors from the design and conceptual phase of the RASPRO-PAF system. In order to provide hands on training, acceptability and experience in the use of the data analytics techniques, we also aim to introduce these to all the practitioners as part of the annual continuing medical education (CME) program.

**Challenges and drawbacks:** One of the major drawbacks of any severity detection and summarization technique is the risk of missing important data. In RASPRO technique, we try to mitigate some of these risks by providing a graded information flow from the multiple sensors to the doctors. The alerts are calculated based on patient and disease specific quantization and threshold levels. Hence, the chances of generation of unnecessary alerts are low. One the other hand, upon receiving these alerts the doctors can further request for detailed data on demand (DD-on-D), using which the doctors can see actual sensor values, the calculated motifs, the frequency maps, as well as any other machine learning based assistive diagnosis. This provides the flexibility to doctors and emergency responders to obtain complete view of the patient condition before deciding any intervention. However, any such system is also fraught with the danger of system failures that could jeopardize the patient’s life, though this could be overcome to a large extent by developing robust hardware and fail-safe firmware. We are also aware that a thorough cost-risk-benefit analysis needs to be carried out before any wide scale deployment. Apart from these, in developing countries there are implementation gaps that need to be addressed which include: (a) intermittent and unreliable mobile connectivity in rural regions, (b) capturing and transmission of data while the patient is mobile, (c) power management in edge devices such as mobile phones to ensure timely processing and transmission of data, (d) whether to do the RASPRO-PAF processing at the edge or in the cloud, and (e) efficient management of remote patient monitoring through educating the support staff in hospitals.

## Conclusion

In this paper, we have reported on the successful design, development, and deployment of a set of 3P tools for healthcare data analytics, called RASPRO-PAFs that transform voluminous physiological sensor data into meaningful motifs using personalized disease severity levels. These motifs have been found to be as effective as, or in many cases better than, the raw sensor data in identification and prediction of critical conditions in patients. Through a step-wise precision process, the doctors can gain further insight into the medical condition of the patient, progressively using quantized symbols, motifs, frequency maps, and machine learning. Furthermore, the criticality of a patient is analyzed from these motifs using a novel interventional time relationship that helps doctors prioritize their time more efficiently. Together, the 3P PAFs helps in personalized, precision and preventive diagnosis of the patients. We have also clinically validated the efficacy of the system using both doctor feedback from the hospital as well as using machine learning techniques. Given the initial acceptance of this tool among the medical community, we are preparing for testing and evaluation in other medical domains as well as large-scale field deployment in global health scenario.

## Additional files

Additional file 1 Moving Window OTS Vs. QTS. The figure shows the F1-score while using a moving window of size 30 mins with varying backward offset from t0. The results show that QTS is always better than OTS in classifying a given window as predictor for AHE or not. (PNG 28 kb)

Additional file 2 Moving Window QTS (B=5) Vs. MTS. The figure shows the F1-score comparison of QTS with B=5 and MTS while using a moving window of size 30 mins with varying backward offset from t0. The results show that MTS is better than QTS except in two time slots. (PNG 21 kb)

Additional file 3 Moving Window QTS (B=10) Vs. MTS. The figure shows the F1-score comparison of QTS with B=10 and MTS while using a moving window of size 30 mins with varying backward offset from t0. The results show that MTS is better than QTS except in two time slots, and also W=10 and W=15 are better summarization windows. (PNG 22 kb)

Additional file 4 Moving Window QTS (B=15) Vs. MTS. The figure shows the F1-score comparison of QTS with B=15 and MTS while using a moving window of size 30 mins with varying backward offset from t0. The results show that MTS is better than QTS except in two time slots, and also W=10 and W=15 are better summarization windows. (PNG 21 kb)

Additional file 5 Moving Window QTS (B=20) Vs. MTS. The figure shows the F1-score comparison of QTS with B=20 and MTS while using a moving window of size 30 mins with varying backward offset from t0. The results show that QTS is marginally better than MTS in four time slots. (PNG 22 kb)

## List of Abbreviations

3P: Precision, presonalization, and prevention
ABP: Arterial blood pressure
AHE: Acute hypotensive episode
AIMS: Amrita institute of medical science
AMI: Alert measure index
BP: Blood pressure
CM: Consensus motifs
CAM: Consensus abnormality motif
CNM: Consensus normality motif
DD-on-D: Detailed data on demand
DL: Deep learning
ECG: Electrocardiogram
HIS: Hospital information system
HR: Heart rate
HRV: Heart rate variability
ICU: Intensive care unit
IoT: Internet of things
IPSO: Improved particle swarm optimization
LSTM: Long short term memory
MAP: Mean arterial pressure
MIMIC: Multiparameter intelligent monitoring in intensive care
ML: Machine learning
MTS: Motif time series
NBP: Non-obtrusive blood pressure
OTS: Original time series
PAF: Physician assist filter
PSM: Patient specific matrix
QTS: Quantized time series
RASPRO: Rapid active summarization for effective PROgnosis
RMSE: Root mean squared error
RRV: Respiratory rate variability
SFM: Severity frequency maps
SpO2: Peripheral capillary oxygen saturation
SVM: Support vector machine
