# Supplementary figures and images for: Data to diagnosis in global health: a 3P approach
Source: BMC Med Inform Decis Mak. 2018 Sep 4;18:78. doi: 10.1186/s12911-018-0658-y (PMC6124014; doi:10.1186/s12911-018-0658-y)

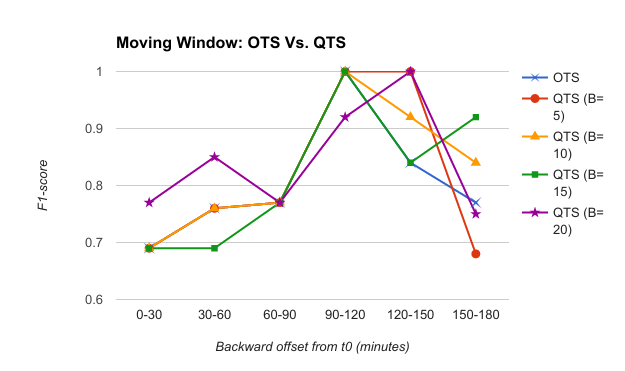

Supplement: Supplementary file 1 — Moving Window OTS Vs. QTS. The figure shows the F1-score while using a moving window of size 30 mins with varying backward offset from t0. The results show that QTS is always better than OTS in classifying a given window as predictor for AHE or not. (PNG 28 kb) [file 12911_2018_658_MOESM1_ESM.png]

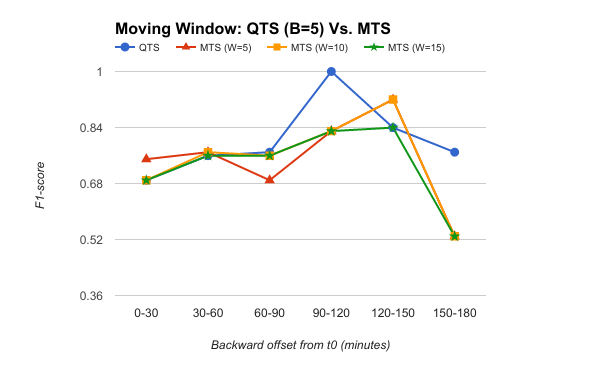

Supplement: Supplementary file 2 — Moving Window QTS (B=5) Vs. MTS. The figure shows the F1-score comparison of QTS with B=5 and MTS while using a moving window of size 30 mins with varying backward offset from t0. The results show that MTS is better than QTS except in two time slots. (PNG 21 kb) [file 12911_2018_658_MOESM2_ESM.png]

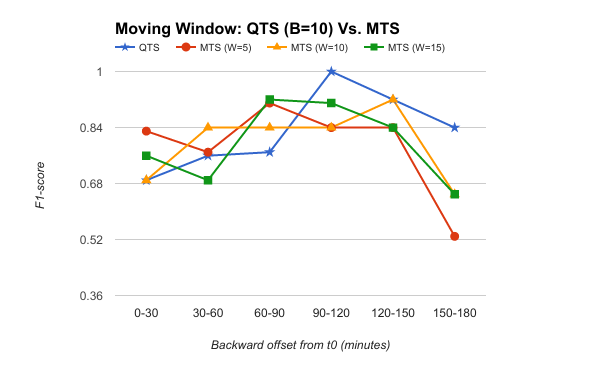

Supplement: Supplementary file 3 — Moving Window QTS (B=10) Vs. MTS. The figure shows the F1-score comparison of QTS with B=10 and MTS while using a moving window of size 30 mins with varying backward offset from t0. The results show that MTS is better than QTS except in two time slots, and also W=10 and W=15 are better summarization windows. (PNG 22 kb) [file 12911_2018_658_MOESM3_ESM.png]

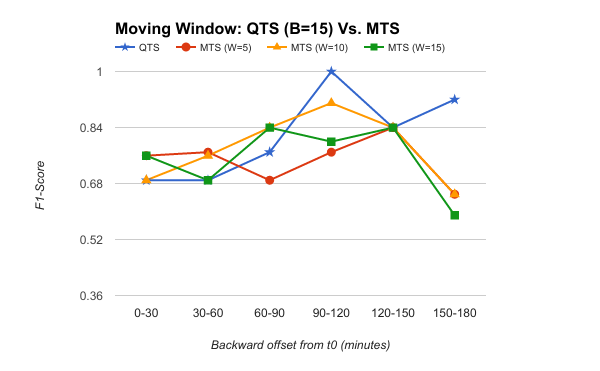

Supplement: Supplementary file 4 — Moving Window QTS (B=15) Vs. MTS. The figure shows the F1-score comparison of QTS with B=15 and MTS while using a moving window of size 30 mins with varying backward offset from t0. The results show that MTS is better than QTS except in two time slots, and also W=10 and W=15 are better summarization windows. (PNG 21 kb) [file 12911_2018_658_MOESM4_ESM.png]

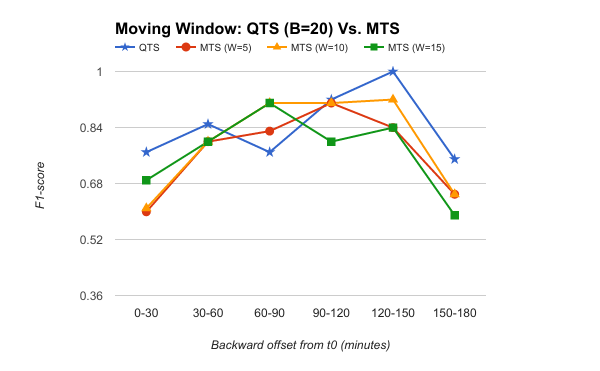

Supplement: Supplementary file 5 — Moving Window QTS (B=20) Vs. MTS. The figure shows the F1-score comparison of QTS with B=20 and MTS while using a moving window of size 30 mins with varying backward offset from t0. The results show that QTS is marginally better than MTS in four time slots. (PNG 22 kb) [file 12911_2018_658_MOESM5_ESM.png]
